# Cultural Challenges Using Technology Following the Kermanshah Earthquake, Western Iran November 2017

**Published:** 2019-08

**Authors:** Ali SOROUSH, Mozhgan SAEIDI, Saeid KOMASI

**Affiliations:** 1. Lifestyle Modification Research Center, Imam Reza Hospital, Kermanshah University of Medical Sciences, Kermanshah, Iran; 2. Cardiac Rehabilitation Center, Imam Ali Hospital, Kermanshah University of Medical Sciences, Kermanshah, Iran; 3. Clinical Research Development Center, Imam Reza Hospital, Kermanshah University of Medical Sciences, Kermanshah, Iran

## Dear Editor-in-Chief

Natural disasters such as floods, storms, volcanoes, and earthquakes are inevitable events that sometimes affect some parts of around the world (1). Recently earthquake in Sarpol-e-Zahab City of Kermanshah, western Iran led to significant financial and financial losses. The severity of the damage was so much that many buildings were destroyed and water, electricity, gas services, and fixed telephone lines were generally disrupted. While people have been reported to be discontent with the use of mobile phones during an earthquake (2), access to the earthquake-stricken Internet users was not a problem, and most people tracked the incident on the first hours of the earthquake via mobile phone. Due to the occurrence of the event at night and the region’s electricity outage, as well as the shock of survivors of the incident, they were following the news about the arrival time of rescue forces, the reunification of the region’s electricity, the statistics of the deaths and injuries, and the likelihood of future earthquakes through mobile phones. One of the most commonly used applications among the disaster survivors was the telegram that the news was quickly transmitted through it.

Mobile and related applications have a significant role to play in crisis management (1, 2). At the time of the crisis, getting news about the event will somewhat lessen the confusion of the victims’ families (3). Nevertheless, in line with previous reports (3, 4) on many sites and the Telegram channels rumors were prevailed on the documentary news. Unrealistic and exaggerated news of the number of victims and injured people, lack of timely relief by rescue forces, a severe drop in temperature and extreme rainfall, and the possibility of more severe earthquakes exacerbated the survivors’ concerns. Many opportunist people were looking to increase their followers or do it for other purposes (4). In this particular case, technology not only did not contribute to reducing the suffering of survivors, but the cultural weaknesses in its use disrupted the mental security of them. This false news and rumors continued, even after the establishment of survivors into tents and metal chambers and raised their concerns.

This dilemma may also be repeated in the country’s future disasters; we recommend that survivors do not have access to cyberspace for at least a few days. On the other hand, the Ministry of Health and educational systems of the country try to eliminate this cultural weakness through appropriate education for different classes of society. Otherwise, at the time of any acute crisis in the country, the psychological crisis caused by cultural problems will be added to other challenges.
